# Emerging trends in biomaterials for sustainable food packaging: A comprehensive review

**DOI:** 10.1016/j.heliyon.2024.e24122

**Published:** 2024-01-04

**Authors:** Md. Zobair Al Mahmud, Md Hosne Mobarak, Nayem Hossain

**Affiliations:** Department of Mechanical Engineering, IUBAT-International University of Business Agriculture and Technology, Bangladesh

**Keywords:** Biomaterials, Food packaging, Biodegradable, Composites

## Abstract

This comprehensive review investigates a variety of creative approaches in the field of sustainable food packaging biomaterials in response to growing environmental concerns and the negative effects of traditional plastic packaging. The study carefully looks at new developments in biomaterials, such as biodegradable polymers, ceramics, composites, and metal alloys, in response to the growing need for environmentally suitable substitutes. It highlights how they might replace conventional plastic packaging and lessen environmental damage. Moreover, the incorporation of nanotechnology into packaging is closely examined due to its crucial function in improving barrier qualities, introducing antimicrobial properties, and introducing smart packaging features. The investigation includes edible coatings and films made of biodegradable polymers that offer new sensory experiences in addition to prolonging the shelf life of products. The review emphasizes the use of biomaterials derived from food processing and agricultural waste, supporting environmentally responsible methods of producing materials while simultaneously using less resources and waste. As a strong defense against plastic pollution, the report highlights the food industry's increasing use of recyclable and biodegradable packaging, which is in line with the concepts of the circular economy. A movement in consumer tastes and regulatory pressures toward sustainable food packaging is evident in global market patterns. Notwithstanding these encouraging trends, there are still issues to be resolved, including cost-effectiveness, technological constraints, and the scalability of biomaterial production. This thorough analysis concludes by highlighting the critical role biomaterials have played in guiding the food industry toward sustainability and emphasizing the need for ongoing research and development to adequately address environmental issues on a worldwide scale and satisfy the growing demand for environmentally friendly packaging options. Biomaterials show great promise as catalysts for the food industry's transition to a sustainable future.

## 1 Introduction

In recent years, there has been an increasing awareness and concern about the environmental effect of traditional food packaging materials across the world [1,2]. The hunt for environmentally acceptable and sustainable alternatives has resulted in an increase in research and innovation in the field of biomaterials for food packaging [3]. This in-depth examination digs into new trends in biomaterials for sustainable food packaging, putting light on the varied range of materials, technology, and market dynamics that are influencing the future of this crucial sector. Exploration of diverse biomaterial categories that have enormous promise for sustainable food packaging is one of the key emphasis topics. Biodegradable polymers, which disintegrate spontaneously over time, have received a lot of interest because of their potential to minimize plastic waste [4]. Ceramics are also being investigated as feasible options for food packaging applications because to their natural durability and resilience to environmental conditions [5]. Composites, which blend several biomaterials to exploit their distinct features, provide a diverse approach to packaging solutions [6]. Furthermore, the use of metals and alloys in food packaging is gaining popularity since they provide strong protection and can be recycled easily [7].

**Fig. 1:**
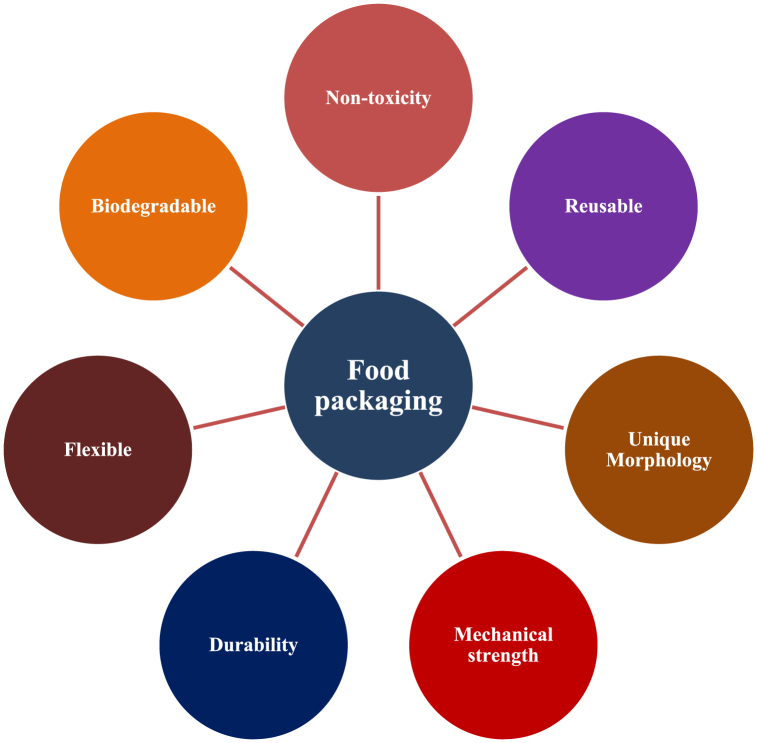
Advantages of biomaterials for food packaging.

Nanotechnology, an innovative field of study, is making considerable inroads into the packaging business [8]. This paper delves deeply into nanotechnology in packaging, emphasizing its potential to increase biomaterial performance, improve barrier qualities, and lengthen food product shelf life. Edible films and coatings, a subset of nanotechnology, represent an intriguing new direction in sustainable packaging, providing novel solutions that not only preserve food but can also be consumed, minimizing packaging waste [9,10]. To present a complete picture, this review digs into worldwide market trends in biomaterials for sustainable food packaging. It investigates the growth trajectories of various biomaterials and technologies, highlighting the places where these breakthroughs are gaining popularity. It also looks at consumer preferences and regulatory frameworks that are altering the packaging environment. Fig. 1 illustrates the advantages of using biomaterials for food packaging.

It is critical to recognize that the move to sustainable food packaging is not without difficulties. The difficulties faced by academics, creators, and policymakers, such as monetary issues, technical constraints, and the requirement for standardized testing and certification. Finally, it provides a glimpse into the field's potential future paths by picturing a situation in which biomaterials play a significant role in minimizing the environmental impact of food packaging. Researchers, businesspeople, and legislators who wish to comprehend the dynamic and evolving world of biomaterials for sustainable food packaging will find this in-depth examination to be a helpful resource. By studying the many types of biomaterials, nanotechnology applications, waste-derived solutions, global market trends, and the challenges and opportunities that lie ahead, it gives a thorough insight on one significant part of the sustainable packaging revolution [11].

The pursuit of sustainable alternatives is being driven by the growing global concern over the environmental impact of existing food packaging materials. Biomaterials are the main emphasis, which is encouraging further research and development in the field of food packaging [12]. This topic includes biodegradable polymers, ceramics, composites, metals/alloys, and other biomaterial categories with intriguing future applications. Interestingly, nanotechnology becomes a key player, increasing the performance of biomaterials, strengthening their barrier properties, and prolonging the shelf life of food products. The talk explores edible coatings and films as fascinating applications of nanotechnology that provide ways to cut down on packaging waste while preserving food. The analysis goes beyond materials to look into global biomaterials industry trends for environmentally friendly food packaging. The development paths of different biomaterials and technologies are examined, paying particular emphasis to how consumer preferences and legal frameworks influence the packaging industry. Fig. 1 provides a graphic representation of the benefits of using biomaterials in food packaging. The introduction notes that despite the clear advantages, there are still obstacles that academics, artists, and legislators must overcome. These obstacles include lack of funding, technical difficulties, and the requirement for certification and standardized testing. A preview of future situations where biomaterials greatly reduce the environmental impact of food packaging is provided in the conclusion. Researchers, industry experts, and legislators looking for insights into the dynamic and developing field of biomaterials for sustainable food packaging may find this thorough analysis to be a useful resource [13].

## 2 Functions of biomaterials

**Fig. 2:**
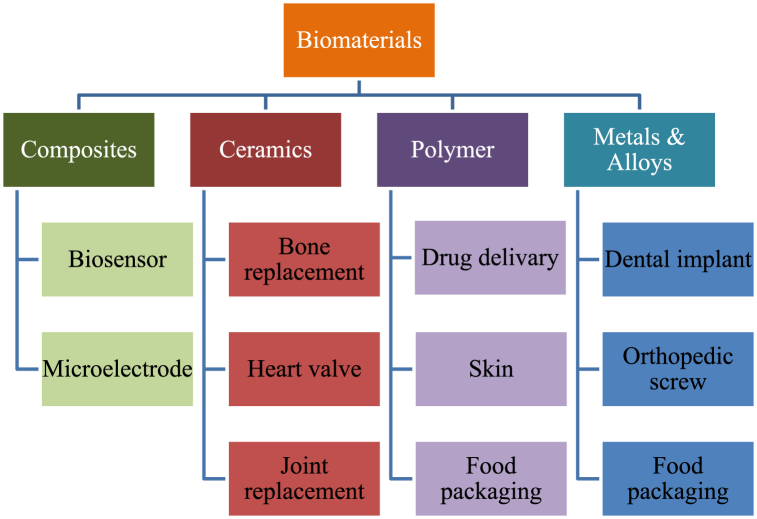
Different types of biomaterials and uses.

Biomaterials have become essential in many areas of business and healthcare, and food packaging is one such area where they have had a big influence [14]. Biomaterials are the best choice for assuring the safety and quality of food items along the whole supply chain due to their wide range of features [15]. Different biomaterials are used in food packaging to satisfy certain needs and each one has its own benefits as shown in Fig. 2. Amazingly, metals and alloys have been used in food packaging, where their strength and longevity assure the safety of consumables, orthopedic screws, dental implants, and even in food [16]. On the other hand, polymers are adaptable biomaterials utilized for prosthetic skin, medicine delivery systems, and food packaging due to their light weight and flexibility [17]. Ceramics are used to protect food goods and have been used in bone replacements, heart valves, and joint replacements because to their biocompatibility. Composites, which incorporate several biomaterials, have also been successfully used in biosensors and microelectrodes, highlighting its relevance in improving food packaging solutions [18]. This broad range of biomaterials serves as an example of the creative steps being taken to develop food packaging technology in order to create a safer and more environmentally friendly future. Below is a list of several biomaterial kinds.

### 2.1 Biodegradable polymers

The increased concern about plastic pollution and environmental sustainability has led to the emergence of biodegradable polymers as a viable biomaterial for food packaging applications. These polymers have a number of benefits and are frequently made from renewable resources like corn starch, potatoes, or sugarcane. When disposed of, they gradually decompose into harmless components, decreasing landfill waste while effectively shielding food goods from environmental variables like moisture and oxygen. Food packaging that decomposes also satisfies rising customer demand for environmentally friendly options while extending the shelf life of food. But there are still issues, such achieving the appropriate amount of barrier characteristics and cost-effectiveness, scalability, and scaleability [19]. Their natural capacity to decompose reduces the negative effects on the environment and addresses issues related to plastic waste. However, there are still issues that need to be resolved through continued study, such as inferior mechanical strength and barrier qualities when compared to traditional plastics. Strengthening formulations can be achieved by mixing or adding reinforcing ingredients. Optimizing processing methods can also lessen these restrictions. In the search for environmentally friendly and sustainable food packaging solutions, biodegradable polymers present a potential material since they strike a compromise between biodegradability and performance [20].

### 2.2 Ceramics

Ceramics have been known as a suitable biomaterial for food packaging, due to their distinctive mix of characteristics. Since these substances are chemically inert and do not interact with food, the flavor and quality of the meal are preserved. Additionally, ceramics have remarkable temperature stability, making them appropriate for both hot and cold food items. Their strong mechanical strength and resistance to damage help make packing more durable and lower the danger of infection. Additionally, ceramics are non-porous, limiting the transport of moisture or gases, extending the shelf life of perishable goods. Because of this, ceramics are becoming a more appealing alternative when looking for sustainable and food-safe packaging solutions [21]. These materials provide strong packaging options because of their remarkable strength and rigidity. They are usually made of oxides, nitrides, or carbides. Because of their exceptional heat stability, ceramics guarantee that food quality will not be compromised while being stored or transported. Their brittleness, however, can be a drawback, requiring cautious handling and design considerations to prevent breaking. Notwithstanding this disadvantage, ceramics' natural strength makes them a good choice for applications involving protective packaging. Using ceramics to its full potential in environmentally friendly food packaging is consistent with the overarching objective of lessening the impact on the environment and encouraging eco-friendly substitutes [22].

### 2.3 Composites

Due to their distinctive mix of qualities, composites are being used more and more as biomaterials in the field of food packaging. These materials generally consist of a matrix, which is frequently a biodegradable polymer like PLA (polylactic acid), reinforced with natural fibers like cellulose or nanomaterials like graphene. This interaction produces packaging materials that are not only sturdy and light in weight but also have good barrier characteristics that stop the passage of oxygen, moisture, and other pollutants, hence increasing the shelf life of food products. Additionally, because composites are frequently biodegradable and have a lower environmental effect than standard plastics, their usage in food packaging is in line with sustainability objectives. Therefore, the search for more efficient and environmentally friendly food packaging solutions points to composites as a viable direction [23,24]. When compared to conventional packaging, their advantages include better barrier qualities, flexibility, and a lower environmental effect. However, there are drawbacks, such as difficulties with recycling because of different material compositions. Furthermore, some composites might not be able to tolerate very high or low temperatures, which could affect which foods they can be used with. Optimizing composite materials for food packaging requires finding a balance between strength, environmental effect, and recyclability. This will ensure both sustainability and functionality in the ever-changing packaging solutions market.

### 2.4 Metal and alloys

Metal and alloys have carved out a space for themselves in the world of biomaterials for use in food packaging because of their distinct features. For instance, stainless steel is highly regarded for its ability to resist corrosion and durability, making it a perfect material for machinery used in food processing and packaging. On the other hand, because of their superior barrier qualities that guard against moisture, light, and oxygen, aluminum alloys are frequently used in the manufacturing of lightweight, recyclable food packaging containers, such as cans and foil. Due to their capacity to be recycled, these materials are crucial parts of contemporary food packaging solutions since they not only guarantee the preservation of food quality and safety but also support sustainability initiatives [25,26].

Understanding the various biomaterial types—such as metal alloys, ceramics, composites, and biodegradable polymers—in detail is essential to understanding the benefits and distinctive characteristics of using biomaterials for sustainable food packaging. For example, naturally disintegrating biodegradable polymers lessen plastic pollution and promote environmental sustainability [27]. Because of their strength and thermal stability, ceramics provide the best possible food preservation. Composites combine several materials to create synergistic effects that improve performance. Metal alloys offer sturdy substitutes because of their strength and malleability. Examining the role of nanotechnology reveals a transformative aspect. The incorporation of nanoparticles into biomaterials enhances their functions by introducing enhanced mechanical strength, barrier qualities, and antibacterial characteristics. The combination of biomaterials and nanotechnology increases the overall effectiveness of sustainable food packaging and opens up new avenues for research into environmentally friendly solutions [28]. As environmental worries over traditional plastic packaging grow, this review critically looks at sustainable alternatives. The environmental risks associated with conventional plastics have prompted a detailed investigation of various biomaterials, nanotechnology, and novel alternatives such as edible films. Making this change is essential to reducing the harm that plastic trash does to ecosystems. In order to protect the environment, the analysis emphasizes how urgent it is to embrace sustainable techniques and materials. A variety of biomaterials, such as biodegradable polymers, durable ceramics, and creative composites, demonstrate encouraging progress in minimizing environmental damage. 10.13039/100014337Furthermore, the use of nanotechnology presents a paradigm change, supporting the functionalities of biomaterials with increased mechanical strength, improved barrier qualities, and improved antibacterial qualities. The investigation of edible films highlights the complex strategy for environmentally friendly food packaging even more.

### 2.5 Nanomaterials

The use of nanomaterials in food packaging has grown in popularity because of their special qualities, which improve packing efficiency and lengthen food product shelf life [29]. When compared to conventional packaging materials, nanocomposites—such as nanoclay and graphene-based materials—offer better mechanical strength, barrier qualities, and thermal stability. Because of their antibacterial qualities, silver nanoparticles lower the risk of foodborne infections by preventing the growth of bacteria and fungi. Because titanium dioxide nanoparticles may block UV rays, they can prevent food from deteriorating due to light. They also add to the features of enhanced barrier. An environmentally beneficial substitute is provided by nanocellulose, which is made from plant fibers and is renewable and biodegradable. Because of its excellent flexibility and tensile strength, it can be used in a variety of packaging applications. Although less prevalent, quantum dots are being investigated for their potential to indicate food freshness through color changes in intelligent packaging. Nanomaterial safety is still a source of concern despite its benefits. The goal of current research and regulatory frameworks is to mitigate any possible dangers related to the release of nanoparticles into food. The necessity for striking a compromise between improving packaging functionality and guaranteeing food safety for consumers is highlighted by the comparative examination of these nanomaterials [30].

Biomaterials are a wide class of materials with distinctive properties that are essential to the field of medical and biotechnological breakthroughs. The nature of their occurrence or origin, dimensional stability, contact with live body tissues, biodegradability, structural aspect, and use are some of the major factors that determine how they should be classified. Researchers and practitioners can build medical devices, implants, and therapeutic treatments by comprehending and classifying biomaterials according to these characteristics. This categorization system enables a thorough investigation of the characteristics and uses of biomaterials, fostering innovation in biotechnology and healthcare while guaranteeing the use of biomaterials in a safe and effective manner. Table 1 displays several biomaterials' categorization schemes.

**Table 1:** Different biomaterials basis of classification [31].

Sl. No.	Basis of Classification	Biomaterials of Different Classes
1	Chemical Composition	Ceramic materials, polymeric materials, metals, and composite materials
2	Nature of Occurrence or Origin	Natural, Semisynthetic, synthetic
3	Dimensional Stability	Nano, Micro, and macro form of biomaterials
4	Interaction with Living Body Tissues	Resorbable, Non-Resorbable, bioactive, and bio-inert
5	Property of Biodegradability	Biodegradable and Biostable
6	Structural Aspect	Porous and Non-porous
7	Application	Diagnostic, Therapeutics, restorative, preventive, and regenerative
8	Application Sites	Intra-corporeal and Extra-corporeal
9	Contact Time with Body Tissue	Limited, Prolonged, and Permanent

The categorization of biomaterials according to several parameters offers useful insights into their properties and uses. This categorization system is primarily intended for use in the medical and biotechnology fields, but it may also be used to the field of food packaging due to some shared material characteristics and interactions with living things [32]. The classification of biomaterials according to their chemical makeup emphasizes their variety, which includes ceramics, polymers, metals, and composites [33]. This diversity is especially important in the context of food packaging since diverse materials have unique benefits. For instance, metals like aluminum are favoured for their barrier qualities whereas polymers like polyethylene are frequently utilized because to their low weight and flexibility [34]. Food packaging materials are influenced by the classification of their type of occurrence or origin (natural, semisynthetic, synthetic). Although synthetic materials like plastics have become more popular due to their adaptability and affordability, natural materials like paper and cardboard have long been utilized.

The same principles of dimensional stability that divide biomaterials into nano, micro, and macro forms may be used to classify the materials used in food packaging. The barrier qualities and shelf life of food items can be improved by using nanomaterials like nanoparticles or nanocomposites [35]. Food packaging may not directly relate to biomaterials' classification as resorbable, non-resorbable, bioactive, or bio-inert based on their interactions with live human tissues. The idea of bioactivity may be used to packing materials that work with food to keep it fresher longer or avoid spoiling, though.

Packaging for food as well as biomaterials both benefit from the trait of biodegradability. In line with the rising need for environmentally friendly packaging solutions, biodegradable packaging materials like PLA (polylactic acid) provide eco-friendly alternatives to conventional plastics [36]. Porous or non-porous materials, structural elements, and other factors can all be utilized to food packaging [37]. Controlled gas exchange is possible using porous materials, which is essential for maintaining the quality of some food items. Applications for biomaterials in the medical industry include diagnostics, treatments, restoration, prevention, and regeneration. Similar to other types of packaging, food packaging fulfills a variety of purposes, including convenience, branding, and preservation. Though not directly applicable to food packaging, the classification's consideration of application sites (intra-corporeal and extra-corporeal) and contact times with body tissue (limited, prolonged, and permanent) highlights the significance of knowing the precise requirements and interactions of materials with their intended environment. In conclusion, the categorization of biomaterials may be used to the field of food packaging even if it was initially created for medical applications. This research shows how biomaterial categorization concepts may be used to choose packaging materials that are suitable for preserving and safeguarding food goods while taking consumer safety and environmental sustainability into account.

The list of biomaterials as shown in Table 2 That might be used in food packaging includes both terrestrial and aquatic sources. Although potatoes, tomatoes, sugarcane, turmeric, jackfruit, maize, and other biodegradable plants present interesting alternatives, their availability and scaleability require evaluation. Citrus peels could contain natural antioxidants, however the effectiveness of the extraction method needs to be considered. Red seaweed has the capacity for plentiful growth and biodegradability, however its usefulness in packaging applications has to be investigated. Although promising for chitosan extraction, using crab shells extensively may provide difficulties. In the end, these biomaterials provide environmentally benign options, but their use in food packaging will depend on successful extraction, scalability, and affordability [38].

**Table 2:** Sources and uses of green-synthesized biomaterials [39].

Source	Extract	Composites	Properties	Application	Reference
Tomato	Lycopene	Whey protein-Sodium alginate-Lycopene	Improved bioaccessibility	Stabilizer	[40]
Sugarcane	bagasse	Nanocellulose-Triethyl citrate-Frankincense	Antibacterial, antioxidant	Pathogen control	[41]
Turmeric	Curcumin	PLA-Curcumin	Antibacterial, antioxidant	Pathogen control	[42]
Jackfruit	Starch	Starch-PVA-ZnO	pH sensing	Packaging	[43]
Citrus	Pectin	Pectin-marjoram oil	Antimicrobial	Active food packaging	[44]
Citrus	Pectin	Pectin-Clove oil	Shelf life, antibacterial	Edible coating	[45]
Red seaweed	Carrageen	Alginate-Carrageen	Flavors, antimicrobials, antioxidants, colors	Edible film coating	[46]
Sugarcane	bagasse	Nanocellulose-Glycerol	Biodegradable	Packaging	[47]
Potato	Cellulose	Cellulose-Sodium alginate-CuO	Antimicrobial, antioxidant	Active packaging	[48]
Corn	Starch	Starch-PVA-Citric acid	Antibacterial	Active packaging	[49]
Grapefruit	Seed TPS	PE-PLA-TPS	Antibacterial	Packaging	[50]
Crab shell	Chitin	Chitin-PVA	Excellent Barrier	Packaging	[51]
Crab shell	Chitin	Chitin eCNF	Biodegradable, biocompatibility	Packaging	[52]
Citrus peel	Pectin	Pectin e Alginate	Antibacterial	Packaging	[53]
Citrus	Pectin	Pectin e Glycerol-CMC	Thermal stability	Packaging	[54]

Natural materials have been used into a variety of food packaging technologies [55]. Packaging made of nanocellulose and sugarcane bagasse is sustainable and biodegradable [56]. Lycopene from tomatoes increases bioaccessibility in packaged foods and serves as a stabilizer. Antibacterial and antioxidant qualities are added to PLA packaging with turmeric, improving infection control [57]. ZnO encapsulation and jackfruit-based starch allow pH sensing capabilities. Packaging made of citrus pectin that also contains marjoram or clove oil has antibacterial properties and increases shelf life. As edible film coatings, carrageenan and alginate from red seaweed provide tastes, antibacterial properties, antioxidant properties, and colors. Excellent barriers, biodegradability, and biocompatibility are offered by chitin produced from crab shells and eCNF-based packaging [58]. Together, these developments propel practical and ecological food packaging solutions.

## 3 Nanotechnology in packaging

Nanotechnology has made great progress in transforming the realm of packaging by integrating biomaterials into its design and manufacturing processes [59]. This novel method combines the special qualities of nanoparticles with the biomaterials' sustainability and biocompatibility to provide packaging solutions that are both useful and ecologically responsible. The application of nanoscale materials including nanoparticles, nanocomposites, and nanofibers in packaging is a crucial component of nanotechnology [60]. These materials enhance the shelf life and safety of packaged goods by providing greater strength, barrier qualities, and antibacterial capabilities. Additionally, packaging that is smart and sensitive that can sense and react to environmental changes like changes in temperature or moisture may be made using nanoscale materials. Nanotechnology enables fine control over material characteristics and may be adjusted to satisfy specific packaging needs such as oxygen or moisture barrier requirements [61]. This level of personalization guarantees that packaging options are suited for the preservation and protection of varied items. The use of nanotechnology and biomaterials in packaging is a cutting-edge solution that solves both functional and environmental concerns [62]. It has the ability to minimize waste, improve product safety, and promote sustainable packaging solutions in a variety of sectors.

Table 3 mentioned reflect a diverse spectrum of advancements in food packaging and storage technologies that make use of nanoparticles to improve performance. While these developments present exciting prospects, a rigorous study is required to assess their consequences. Products like Debbie Meyer® GreenBags and NanoSealTM coatings, on the other hand, demonstrate environmentally aware attempts to prevent food waste by increasing the shelf-life of perishables. Items impregnated with nanosilver, such as infant milk bottles and food containers, offer improved antibacterial characteristics, which will benefit food safety. Zeomic® silver zeolites packaging film and Agion® technology reveal novel approaches to using silver's antibacterial properties.

**Table 3:** Shows some instances of food packaging products readily accessible in the market that incorporate engineered nanomaterials (ENP) [63].

Company	Brand/Product	Description
Debbie Meyer® Innovation (U.S.)	Debbie Meyer® GreenBags	Food storage bags containing nano-clay to preserve freshness and extend the shelf-life of fruits and vegetables.
Baby Dream Co. Ltd® (Korea)	Nano-silver Baby Milk Bottle	Baby milk bottle containing nanosilver for antimicrobial properties.
NanoPack Inc. ® (U.S.)	NanoSeal™ - Barrier Coating and NanoSeal™ - Bairicade XT™ Barrier Coating	Coating applied to traditional packaging films to enhance gas barrier properties, approved for indirect food contact.
Baby Dream Co. Ltd® (South Korea)	Silver-nano Noble one-touch mug cup	Mug cup with silver-nano coating.
Debbie Meyer® Innovation (U.S.)	Debbie Meyer® Bread Bags™	Bread bags containing nano-clay for bread storage.
Colormatrix® (U.S.)	PET bottles with nano-titanium nitride	PET bottles with nano-titanium nitride for barrier properties.
Miller Brewing Co® (U.S.) and Hite Brewery Co.® (South Korea)	Plastic beer bottles with nano-clay	Plastic beer bottles containing nano-clay for barrier properties.
Zeomic Co Ltd® (Japan)	Zeomic® silver zeolites packaging film	Packaging film with silver zeolites for antimicrobial properties.
Oso Fresh® (U.S.)	Fresh food containers with nanosilver	Food containers containing nanosilver particles.
A-DO Global® (South Korea)	Nano-silver food containers	Food containers with nanosilver for antimicrobial properties.
A-DO Global® (South Korea)	Nano-silver NS-315 water bottle	Water bottle with nanosilver coating.
Changmin Chemicals® (South Korea)	Nano-silver salad bowl	Salad bowl with nanosilver coating.
Sharper Image® (U.S.)	FresherLonger™ Miracle Food Storage	Food storage containers containing nanosilver.
Sharper Image® (U.S.)	FresherLonger™ Plastic Storage Bags	Plastic storage bags containing nanosilver.
BlueMoonGoods™® (U.S.)	Fresh box silver nanoparticles food storage containers	Food storage containers with silver nanoparticles.
Kinetic Go Green® (U.S.)	Smartwist food storage with nanosilver	Food storage containers with nanosilver coating.
Pabck® (U.S.)	Clear Silver Reclosable Mylar Zip Lock Bags	Aluminum foil packaging with a clear silver layer.
Honeywell® (U.S.)	Aegis® OX	Oxygen-scavenging barrier resin for PET bottle applications, containing passive nano-clay particles.
Quan Zhou Hu Zeng Nano Technology Co.Ltd® (China)	Nano-silver storage box	Storage box with nanosilver coating.
Hopack® (Australia)	Nanobox	Paper food box/container containing nanosilver.
Agion Technologies® (U.S.)	Agion®	Technology containing silver zeolites for controlled release of antimicrobial ions.
SongSing Nano Technology Co. Ltd® (Taiwan)	Nano Plastic Wrap	Plastic wrap containing nanozinc oxide as a light catalyst for sterilization.
Nanocor Inc® (U.S.)	Imperm®	Multi-layer PET bottles and sheets for food and beverage packaging with nano-clay to extend shelf life.
InMat® Inc, U.S.	Nanolok™	Water-based nano-clay-based composite coating with high oxygen barrier for transparent packaging.

However, there are some reservations. The long-term safety of nanoparticles for both human health and the environment is unknown. Because of the possibility of nanoparticle migration into food, there are concerns regarding their safety in direct contact applications. Furthermore, the environmental impact of nanoparticle disposal or release during product degradation should be taken into account. Furthermore, while increasing shelf life is beneficial, it may inadvertently encourage wasteful practices if consumers use it as a crutch rather than tackling bigger food sustainability concerns [63].

These nanotechnology-based packaging solutions have the potential to improve food safety and reduce waste, but they must be thoroughly evaluated in terms of long-term safety, environmental effect, and consumer behavior consequences. Striking a balance between innovation and accountability is critical as these goods gain commercial traction.

## 4 Edible films and coatings

A thin layer of edible substance that is generated as a protective coating on meals and may be ingested together with those items is known as an edible coating. Typically, the product is submerged in a film-forming solution created by the structural matrix before these layers are applied in liquid form to the food's surface. In nature, edible films are free-standing structures, whereas edible coatings cling to the surface of food [64]. In order to create a continuous framework of films or coatings, many bio-based polymers have been researched. The most prevalent class of biopolymers employed in the creation of edible materials are hydrocolloids, which include both polysaccharides and proteins. Sources for them include plants, animals, and microbes. The most widely used polysaccharides in the manufacture of edible films and coatings are cellulose derivatives, starches, alginates, pectins, chitosans, pullulan, and carrageenans, while the most widely used proteins are soybean proteins, wheat gluten, corn zein, sunflower proteins, gelatin, whey, casein, and keratin [65]. However, the nature of such substances is hydrophilic. As a result, various oils and fats are added to hydrocolloid matrix to improve their water vapour barrier qualities. Wax, triglycerides, acetylated monoglycerides, free fatty acids, and vegetable oils are the most often used [66]. Biopolymers have traditionally been used as one-component film or coating formulations, and this trend is still present today. But recently, a lot of research has been done on two- and multi-component edible polymers that offer better functional qualities. To create structures with altered physical, mechanical, and barrier qualities that are superior to the one-component material, composite films or coatings are generated in this context by combining two or more film-forming components. Thus, in film-forming formulations, a variety of compounds are utilized to enhance or change the material's fundamental functioning, such as plasticizers, crosslinking agents, emulsifiers, and reinforcements.

To further enhance the quality, stability, and safety of packaged foods, various active chemicals, including antimicrobials, antioxidants, colorants, flavors, and nutraceuticals, are added to the film-forming solution. Additionally, such components could give edible material antibacterial, antifungal, or antioxidant capabilities [67,68]. Potential uses for innovative edible coatings are shown in Table 4.

**Table 4:** Lists possible uses for innovative edible coatings [69].

Coating Materials	Food Products	Main Advantages	References
Yam starch	Strawberries	Reduced decay, weight loss and firmness	[70]
Gum arabic	Anna apple	Reduced decay	[71]
Almond gum	Sweet cherries	Decrease in respiration rate and ethylene production; delayed the changes in color, weight loss, firmness, titratable acidity and soluble solid concentration	[72]
Gum arabic	Strawberries	Inhibited fungal growth	[73]
Gum arabic	Tomatoes	Inhibited fungal growth	[74]
Apricot gum containing *Satureja intermedia* extract	Wild almond kernels	Lower fungal contamination, oxidative compounds content and fatty acid profile variation	[75]
Gum arabic with lemongrass and cinnamon essential oill	Banana and papaya	Antifungal effect; reduced the growth of *Colletotrichum musae* and *Colletotrichum gloeosporioides*	[76]
Potato peel waste with oregano essential oil	Salmons (Cold-smoked)	Reduced the growth of L. *Monocytogenes*	[77]
*Cordia myxa* gum	Artichoke bottoms	Delayed browning; overall shelf-life extension	[78]
Mucilage extract from *Opuntia ficus-Indica* cladodes	Figs	Maintained the fruit weight and firmness	[79]
*Opuntia cactus* polysaccharides	Kinnow mandarin	Increase shelf life with regard to its ph, acidity, aroma, color, texture and general appearance	[80]
*Aloe vera* Gel	Apple	Maintained the bioactive compounds; reduces weight loss and firmness	[81]
Arabic gum with *Aloe vera* and garlic extract	Guava	Shelf-life extension; higher ascorbic acid content; lower content of total sugars	[82]
Fruit and vegetable residue flour from orange, passion fruit, watermelon, lettuce, courgette, carrot, spinach, mint, taro, cucumber, and rocket	Carrots (fresh-cut)	Delayed weight loss; maintained the color	[83]
Fruit and vegetable residue flour from orange, passion fruit, watermelon, lettuce, courgette, carrot, spinach, mint, taro, cucumber and rocket with the addition of potato peel flour	Acerolas	Delayed weight loss	[84]
Almond gum exudate	Tomatoes	Delayed the changes in color, weight loss, titratable acidity, soluble solid concentration, ascorbic acid content, firmness, and decay percentage	[85]
Almond gum	Bananas (slices)	Delayed the changes in weight loss; lower browning index	[86]

There are several benefits to using different coating materials to preserve and improve the quality of various food products [87]. Strawberries with a yam starch coating have less deterioration, less weight loss, and more firmness, increasing their shelf life. Gum arabic is helpful in preventing fungal development in strawberries and tomatoes as well as reducing deterioration in Anna apples. When applied to sweet cherries, almond gum exhibits a variety of advantages, including a reduced rate of respiration, a reduction in ethylene generation, and a delay in the occurrence of alterations in a number of quality indicators, including color, weight loss, firmness, titratable acidity, and soluble solid concentration. Apricot gum with Satureja intermedia extract also reduces oxidative substances and fungal contamination in wild almond kernels. Gum arabic functions as an antifungal agent for bananas and papayas, reducing the growth of dangerous fungus when combined with lemongrass and cinnamon essential oil. Listeria monocytogenes growth in cold-smoked salmon is significantly reduced by the addition of potato peel waste and oregano essential oil. Cordia myxa gum prevents browning and increases the shelf life of artichoke bottoms.

By maintaining the pH, acidity, fragrance, color, texture, and overall look of Kinnow mandarins, opuntia cactus polysaccharides extend their shelf life. Apples lose less weight and retain more firmness when treated with aloe vera gel, and guavas have a longer shelf life, more ascorbic acid, and fewer total sugars when treated with a mixture of Arabic gum, aloe vera, and garlic extract. In addition, when mixed with potato peel flour for acerolas, fruit and vegetable residue flour from diverse sources prevents weight loss and maintains the color of freshly cut carrots. With a variety of fruit and vegetable products, these coating materials exhibit their exceptional capacities to enhance food quality and shelf life, providing useful solutions for food preservation and waste reduction [69].

The idea of packaging has evolved in the modern era such that packaging systems may now include additional features like antioxidant activity, antimicrobial qualities, oxygen scavenging, and sensor presence. This means that traditional packaging is now known as active and/or intelligent packaging, some of which are edible films or coatings [88,89]. A film is typically thought of as a thin, independent solid sheet that is produced using at least one processing method, applied, and utilized to package or hold food items. Conversely, a liquid coating is directly applied to the surface of food products by brushing, sprinkling, or dipping techniques. The authors would want to make clear that, while film creation occurs in situ, coating the surface of food products, the term “film” is occasionally also used to refer to coatings.

**Fig. 3:**
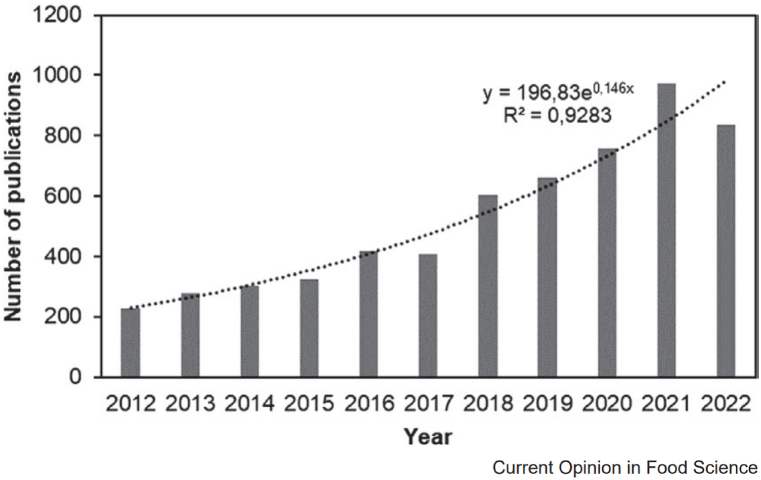
Research pertaining to novel edible coatings [93].

As Fig. 3 shows, the field of study known as “edible films/coatings” has grown tremendously in recent years. Using the online database SCOPUS, the search was restricted to the last 10 years and the keywords “edible,” “coatings,” and “films.” It is evident that there have been around five times as many publications on this subject as there were in 2012. Almost half of all published material in the past 10 years has been published if we limit our analysis to the previous few years (2020–2022). However, food has been preserved and given a longer shelf life for ages thanks to edible coatings and films. Wax or lard applied to fruits, vegetables, meat, and fish are a few examples [90]. Furthermore, investigators have recently investigated a few particular uses for edible films that are connected to their utilization as packaging solutions. These consist of gelatine and soybean polysaccharide soluble sachets for soups and beverages (to be solubilized in water) [91]. Using gelatine-pectin to make edible wrappers that reduce ricotta cheese's moisture content is another example [92]. It's also possible to use edible films in place of the original packaging for individual candies.

## 5 Recyclable and compostable packaging

Biomaterials-based recyclable and biodegradable packaging has become a viable alternative to the traditional single-use plastics that pose environmental problems [94]. These cutting-edge materials are biodegradable and less dependent on fossil fuels since they are made from renewable resources like algae, cellulose, or plant-based starches. By collecting and processing recyclable biomaterial packaging through current recycling systems, a circular economy may be created while reducing waste and saving resources.

On the other hand, compostable biomaterials decompose naturally into organic matter, improving soil and lowering landfill trash [95]. In addition to lowering the carbon footprint of packaging, this strategy promotes a more responsible and eco-friendly approach to product packaging. But for implementation to be successful, there must be broad acceptance, greater consumer awareness, and upgrades to the infrastructure for data gathering and processing. Packaging made of biomaterials that is recyclable and compostable is a potential way to lessen the environmental effect of packaging materials, which is something that consumers and businesses are prioritizing more and more [96].

Table 5 illustrates numerous producers are providing a variety of environmentally acceptable substitutes for conventional plastics, which is quickly broadening the landscape of biodegradable and biobased polymers. While NatureFlex from Innovia Films shines in filmmaking, Mater-Bi, a starch-based product from Novamont, finds uses in loose fill, bags, films, trays, and wrap. Tenite from Eastman offers versatility in film applications, and Biograde from FKuR gives cellulosics a new facet. Strong competitors for PLA, a well-known biopolymer, including BASF's Ecovio, NatureWorks' Ingeo, and Cargill Dow's EcoPLA, with uses in rigid containers, films, and barrier coatings. The need for bottles, trays, and films is satisfied by Dupont's Biomax bio-based PET. As a bio-based substitute for rigid containers, film wrap, and barrier coatings, Braskem's Bio-PE is available. With uses in trays, films, and barrier coatings, Monsanto's Biopol and Biomer products are essential for PHA/PHB. This growing selection of bio-based polymers highlights the industry's dedication to sustainability and provides both producers and customers with a wide range of environmentally responsible options, supporting the worldwide initiative to minimize plastic pollution and environmental damage [97].

**Table 5:** Utilizations of biomaterials that are compostable and recyclable [97].

Polymer Type	Manufacturer	Brand	Applications
Starch	Novamount	Mater-Bi	Loose fill, bags, films, trays, wrap
	Innovia films	NatureFlex	Films
Cellulosics	Eastman	Tenite	Flexible film
	FKuR	Biograde	
PLA	BASF	Ecovio	Rigid containers, films, barrier coating
	NatureWorks	Ingeo	
	Cargill Dow	EcoPLA	
Bio-based PET	Dupont	Biomax	Bottles, trays, films
Bio-based PE	Braskem	Bio-PE	Rigid containers, film wrap, barrier coating
PHA/PHB	Monsanto	Biopol	Films, barrier coating, trays
	Biomer	Biomer	

In commercial applications, starch-based packaging has become more popular, especially in the food packaging sector. Producers such as Paper foam, Bio4Pack, Novamont, and Plantic are leading the way in using this environmentally beneficial material. Transparent films from Novamont and net packaging from Bio4Pack are excellent choices for fruit and vegetable packing since they are sealable, have a great finish, and are durable. Complying with sustainable packaging norms, these materials are compostable and biodegradable (Kabasci, 2020; Molenveld et al., 2015) [98,99]. Plantic offers transparent, oxygen- and water-resistant barrier films that can be coated with glass or aluminum for use in the packaging of meat and fish. These components guarantee the quality and freshness of perishable goods. Plantic's and Amcor's film laminates, respectively, are certified for direct food contact and promote composting and biodegradation in cheese and dry food packaging. Egg cartons (Paperfoam), bread packaging (Biofutura), coffee capsules (Ethical Coffee Company), and candy containers (Cadbury) are examples of products made of starch. These applications exhibit adaptability, featuring features such easy recyclability, lightweight design, and smooth finish. Furthermore, both drinking straws (Moonen Natural) and hot drink plates/cups (Biome Bioplastics) are made of materials that can be heated to a high temperature while still adhering to strict food safety standards [100].

To summarise, starch-based packaging materials are advancing significantly in a range of commercial applications, offering a balance between sustainability, food safety regulations, and practicality. These developments support the current trend toward packaging options that are more responsible and ecologically friendly.

## 6 Global market trends

There has recently been a surge in articles in the literature on the reuse of agricultural and food waste, indicating a large market for high-benefit goods with high economic value. As a result, there is growing interest in agro-food waste as a source of bio-based materials with potential use in the packaging sector. The market for bioplastics is expected to reach USD 2.87 million in 2025, a 36 % increase from 2020, while the market for food packaging was estimated to be worth USD 346.5 billion in 2021 [101,102]. Additionally, the majority of the world's top manufacturers of bioplastics in 2022 will be located in Asia, which will account for more than 41 % of global output, compared to just 26.5 % in Europe, 18.9 % in North America, and 12.6 % in South America [84]. A burgeoning market for bioplastic packaging is being addressed by bio-based materials made from agri-food wastes, which have various advantages in terms of their positive effects on the environment. Utilizing sustainable resources across a material's whole life cycle helps with sustainability. The starch fiber, cellulose fiber, polysaccharides, chitosan, PLA, PHB, and PHA dominate the market for bio-based materials, but additional substances that might be employed as bioactive components are also present, as indicated in Table 6.

**Table 6:** Shows the global market size for bio-based materials [103].

Bio-Based Material	Market Size (USD) (Year)	Reference
Starch fiber	97.85 Bn (2020)	[104]
Cellulose fiber	35.20 Bn (2021)	[105]
Pigment	34 Bn (2020)	[106]
Polysaccharide	12.2 Bn (2018)	[107]
Antimicrobial coating	9 Bn (2021)	[108]
Chitosan	6.8 Bn (2019)	[109]
Antioxidant	3.92 Bn (2020)	[110]
Pectin	944.45 Mn (2021)	[111]
Polylactic Acid (PLA)	698.200 Mn (2020)	[112]
Nanocellulose	291.53 Mn (2019)	[113]
Poly-3-Hydroxybutyrate (PHB)	102.4 Mn (2021)	[114]
Polyhydroxyalkanoates (PHA)	85 Mn (2021)	[115]

The global market for bioplastics is expected to rise from 2.23 million tons in 2022 to 6.3 million tons in 2027, providing a significant potential opportunity. In 2022, food packaging will continue to be the most popular application, accounting for 48 % of the global bioplastics market [101]. The market for cellulose fibers may exceed USD 60.01 billion by 2028 [105], and manufacturing of nanocellulose has been the subject of intense research on a global scale, with the majority of pilot and commercial production facilities situated in industrialized nations. While American Process manufactures 1000 kg/d of CNF, CellForce in Canada set up a CNC pilot production to prepare 300 tons annually. However, only a small number of businesses, like VTT, created a CNF-based plastic film for food packaging using the waste products of a food production process.

The most recent market data gathered by European Bioplastic hows that in 2022, biodegradable plastics comprised more than 51 % of the world's bioplastic output, with PLA accounting for 20.7 % of that and expected to rise to 37.9 % in 2027. Regarding the PLA manufacturing chain, the fermentation processes, raw materials substrate, and lactic acid synthesis account for around 40–70 % of production expenses [116]. The application has an impact on the ultimate price, which now stands at 4.6 USD/kg and typically tracks the cost of the fermentation feedstocks. When pre-treated maize stover was employed as a substrate, a research found that the minimum selling price for lactic acid was 0.56 USD/kg; as a result, using renewable and inexpensive resources enables a more economically viable method [117,118].

Companies are interested in lowering the cost of eco-friendly packaging since its market share has been expanding. The PLA market is anticipated to grow by 26.6 % between 2022 and 2030, with packaging accounting for more than 36 % of sales [119]. With various technical manufacturing methods, the top competitors on the international market for PLA are Total Corbion, NatureWorks, Supla, Futerro, and Cofco [120]. Instead of using materials derived from plants, NatureWorks' technology can use greenhouse gases; Corbion is actively investigating the use of second- and third-generation feedstock, including food waste and industrial waste streams; and Futerro established a new integrated biorefinery in Europe to produce and recycle PLA [121,122].

Similar to the manufacturing of PLA bioplastic, the cost of the raw materials for PHA is expensive (between 30 and 40% of the overall production expenses), reported at USD 2.6/kg when sucrose is used as the carbon source, with a payback period of 2.9 years and a return on investment of USD 34.2 % [123]. When sugarcane bagasse is used as the carbon source to create P3HB, the process can become more economically competitive for an industrial facility, as was discovered, some businesses have successfully implemented this idea, as demonstrated by Bio-on, which used sugar beet byproducts and molasses as raw materials to produce PHB [124]. Genecis and Full Cycle have utilized food waste destined for landfills as raw materials to create biodegradable plastics and other high-value products [125,126].

Businesses have started experimenting with bioplastic options, and a growing number of major names have unveiled their first substantial goods [127]. Agri-food by-products have been proved to be potential raw materials in various biotechnology processes, which are made possible by the collaboration between businesses and academia [128]. Increased availability of bioplastics along with a variety of new materials, goods, and uses on the market have made bioplastics a desirable and well-liked option for customers.

**Fig. 4:**
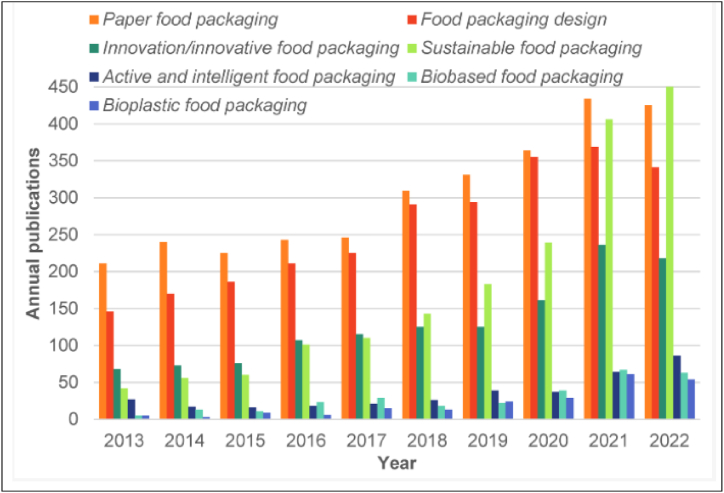
Total number of publications on food packaging that have been published in the literature over the past 10 years [129].

The field of biomaterials for sustainable food packaging has seen a significant upsurge in research effort during the last ten years, as Fig. 4 illustrates. The exponential rise in articles in a variety of journals emphasizes how important it is becoming to use biomaterials, especially for paper-based packaging. Notable developments include creative methods for packaging food, characterized by proactive and thoughtful packaging solutions. The incorporation of bioplastics has become a significant aspect, providing eco-friendly substitutes that lessen the environmental impact of conventional packaging materials. Beyond material composition, innovation includes intricate designs that represent a paradigm shift in the practicality and aesthetics of food packaging. Minimizing environmental footprints through sustainable techniques has been a key point. Biobased packaging materials are becoming more and more popular as effective replacements for traditional materials because they are made from renewable resources. This thorough analysis highlights the diverse development of biomaterials in food packaging and captures the dynamic research and development environment targeted at promoting sustainability and lessening the environmental impact of the packaging sector [129].

## 7 Challenges and future directions

The use of biomaterials for sustainable food packaging faces a variety of difficulties and opportunities, involving a complex interaction of technological, financial, environmental, and governmental issues [130]. Finding the delicate balance between cost-effectiveness and sustainability is one of the biggest challenges we face as we make the move to more environmentally friendly packaging alternatives. Although biodegradable metals, ceramics, composites, and polymers show great promise, there are still significant barriers to their commercial scalability and cost [131]. Additionally, the incorporation of nanotechnology into packaging materials opens up fascinating possibilities for improved barrier qualities, antimicrobial activity, and shelf-life extension, but it also necessitates stringent safety evaluations and regulatory frameworks to guarantee consumer well-being [132]. An intriguing category of biomaterials called edible films and coatings has the potential to drastically cut plastic waste. For their adoption to be successful, improvements in flavor, texture, and functionality are required. An eco-friendly alternative is provided by waste-derived biomaterials, such as those made from post-consumer waste or agricultural leftovers. Nevertheless, overcoming processing obstacles and guaranteeing constant quality are necessary for their development. The sustainable packaging industry must include recyclable and biodegradable packaging materials [133]. To reach their full potential, though, effective infrastructure for collection and recycling is required, along with consumer education and adherence. The need for environmentally friendly packaging choices is rising as a result of rising consumer awareness of sustainability problems.

Overall, there are significant obstacles facing the developing field of biomaterials for environmentally friendly food packaging. First of all, there is a challenge in guaranteeing scalability and cost-effectiveness without sacrificing material performance. Second, removing regulatory obstacles and creating a single framework are necessary to achieve industry-wide adoption and standardization. Furthermore, it is still difficult to balance the various needs of various food products while taking temperature, moisture content, and shelf life into account. In order to successfully integrate biomaterials into the food sector, ensure their viability as a replacement for traditional packaging, and support a more ecologically friendly and sustainable approach, it is imperative that these problems be addressed. Adopting biomaterials for environmentally friendly food packaging presents a number of difficulties. Achieving economies of scale and scalability without sacrificing material performance calls for creative production techniques. In order to get beyond regulatory obstacles, a consistent framework and thorough safety assessments of materials infused with nanotechnology must be established. It's still difficult to strike a balance between the various requirements of various food products while taking shelf life and temperature into account. Improving consumer education to encourage eco-friendly choices, developing infrastructure for effective collection and recycling, and developing processing technologies for waste-derived biomaterials are the ways to find solutions. It is imperative to tackle these obstacles in order to effectively include biomaterials and promote an eco-friendly and sustainable packaging paradigm [134].

In order to stimulate innovation and standardization in biomaterials for sustainable food packaging, cooperation between industry players, research organizations, and governmental agencies will be crucial in the future. To allow their wider acceptance in packaging applications, research efforts should concentrate on enhancing the performance and financial viability of biodegradable metals, ceramics, composites, and polymers. At the same time, careful consideration should be given to the integration of nanotechnology, with a focus on thorough safety evaluations and open labeling to win over customers. The sensory qualities and functionality of edible films and coatings must be improved in order to increase their consumer appeal and suitability for a wider range of food products. Further research should be done on waste-derived biomaterials, with an emphasis on creating scalable and effective manufacturing methods that can transform agricultural waste and post-consumer materials into high-quality packaging options. In order to ensure that these materials can be efficiently collected, processed, and reintegrated into the manufacturing cycle, recyclable and compostable packaging should be promoted. Additionally, consumer education programs ought to encourage ethical disposal and recycling methods. It is crucial to monitor and respond to changing market trends on a worldwide scale, coordinating the development of biomaterials with the changing requirements of diverse sectors [135]. This calls for ongoing market research and the adaptability to deal with new possibilities and difficulties. The worldwide use of sustainable packaging materials will also be facilitated by the harmonization of international norms and laws. Further investigation into bio-based polymers, such PLA and PHA, has the potential to yield packaging materials that degrade naturally. Investigating uses of nanotechnology, such as nanocomposites, can also improve barrier qualities and increase packaged food's shelf life. As the need for food safety and quality assurance grows, smart packaging technologies—such as sensors for in-the-moment freshness monitoring—are being integrated. It is also essential to promote the concepts of the circular economy by emphasizing the recyclable and compostable nature of biomaterials. Examining the economic viability and scalability of production techniques such as enzymatic synthesis or microbial fermentation might promote their widespread use. To ensure the safety of innovative biomaterials and create uniform testing methodologies, cooperation between industry, academia, and regulatory authorities is crucial. Sustainable food packaging will be shaped in large part by adopting a holistic strategy that takes into account every stage of the life cycle, from obtaining raw materials to disposing of waste at the end of its useful life [136].

The difficulties and potential prospects for biomaterials for sustainable food packaging highlight the importance of a comprehensive strategy. Players in the sector, researchers, and legislators will need to work together to overcome the financial, technological, and regulatory barriers. The benefits, however, are significant: less plastic waste, improved environmental sustainability, and a more thoughtful approach to packaging that meets the changing expectations of customers throughout the world. Innovation, education, and a firm commitment to building a more sustainable future for food packaging are the way forward.

## 8 Conclusion

Biomaterials research for sustainable food packaging is a viable area in resolving environmental challenges connected with traditional packaging materials. Metals and alloys, polymers, ceramics, and composite materials containing nanoparticles provide a variety of alternatives for improving packaging sustainability. These materials offer prospects to reduce dependency on nonrenewable resources, reduce pollution, and lower the packaging industry's ecological imprint. To fully exploit the promise of biomaterials in food packaging, it is critical to emphasize research and development while encouraging collaboration among scientists, companies, and legislators. Biodegradability, recyclability, and the use of renewable resources should be prioritized in sustainable packaging solutions. Furthermore, adding cutting-edge technologies like smart packaging and advanced processing processes can improve the performance and functionality of biomaterial-based packaging. It will be critical to educate stakeholders on the environmental benefits of biomaterials and incentivize their adoption through legislative frameworks or market-driven efforts. The packaging sector can make a substantial contribution to a more sustainable and ecologically friendly future by embracing these comprehensive initiatives [135].

## Data availability statement

The data associated with this study has not been deposited into a publicly available repository. However, upon request, the data will be made available to facilitate transparency, peer review, and collaboration. We acknowledge the importance of sharing research data to enable other researchers to evaluate and build upon our findings, fostering trust in the scientific community. Our commitment to data availability reflects our dedication to advancing knowledge and promoting sustainable practices in biomaterials for food packaging.

## Additional information

No additional information is available for this paper.

## CRediT authorship contribution statement

**Md. Zobair Al Mahmud:** Writing - original draft, Investigation, Funding acquisition, Formal analysis, Data curation. **Md Hosne Mobarak:** Writing - review & editing, Methodology. **Nayem Hossain:** Supervision, Writing - review & editing.

## Declaration of competing interest

The authors declare that they have no known competing financial interests or personal relationships that could have appeared to influence the work reported in this paper.
